# Language Dominance and Cognitive Flexibility in French–English Bilingual Children

**DOI:** 10.3389/fpsyg.2018.01697

**Published:** 2018-09-07

**Authors:** Elena Nicoladis, Dorothea Hui, Sandra A. Wiebe

**Affiliations:** Department of Psychology, University of Alberta, Edmonton, AB, Canada

**Keywords:** bilingualism, executive function, cognitive flexibility, translation equivalents, balanced bilingualism

## Abstract

Some studies have reported a cognitive advantage for bilingual children over monolinguals and other studies have not. One possible reason for these conflicting results is that the degree of cognitive flexibility is related to individual differences in language dominance and use. More balanced bilinguals who separate their languages by context might have to learn to reduce inter-language interference and therefore show greater cognitive flexibility. The goal of the present study was to test if language dominance is related to French–English bilingual children’s cognitive flexibility, using three different measures of language dominance: (1) parental reports of dominance, (2) relative scores on vocabulary tests, and (3) knowledge of translation equivalents. We also included two measures of language use: (1) living in a bilingual community (Montreal) or a monolingual community (Edmonton) and (2) language separation. Sixty-two French–English bilingual between 46 and 85 months of age participated. Children’s cognitive flexibility was assessed using the Advanced Dimensional Change Card Sort task. Children’s language knowledge and use was assessed in both French and English using a battery of tests. The results showed that none of the measures of language dominance or language use predicted cognitive flexibility. These results are inconsistent with the claim that individual differences in language dominance and use predict bilinguals’ executive function s.

## Introduction

Even when processing only one language, bilinguals have both languages activated in their minds (Grosjean, 1989; Green, 1998; Costa, 2005; Rodriguez-Fornells et al., 2005). Bilinguals therefore have to constantly control interfering information from their two active and competing language systems in order to select the relevant language and inhibit the other that is not in use at that moment (Rodriguez-Fornells et al., 2006; Abutalebi and Green, 2007; Costa et al., 2009; Festman and Münte, 2012). Some researchers have argued that bilinguals’ experience with selecting and inhibiting languages could generalize to other tasks involving attentional processing and cognitive flexibility (Bialystok, 2001; Bialystok et al., 2005). Cognitive flexibility refers to the ability to shift between mental sets and tasks (Miyake et al., 2000) while selective attention refers to the ability to orient attention toward a specific stimulus while simultaneously ignoring other stimuli (Plude et al., 1994; Mahone and Schneider, 2012). Both cognitive flexibility and selective attention are higher mental functions responsible for goal-directed behavior, or executive functions (EFs; Best and Miller, 2010).

If the experience of selecting and inhibiting languages generalizes to other tasks involving EF, bilinguals might outperform monolinguals on non-linguistic measures of EF. This bilingual advantage might be particularly salient during times of developmental change, like childhood and old age (Bialystok et al., 2006; Craik and Bialystok, 2006). During childhood, there is rapid growth of EFs during childhood due, in part, to the pronounced plasticity and maturation of the prefrontal cortex, which allows children to increasingly control their actions and thoughts (Diamond, 1991, 2002, 2009; Diamond and Taylor, 1996; Lengua et al., 2007; Conway and Stifter, 2012). This sensitive period of development provides an opportunity for some positive contextual experiences, such as socioeconomic status and parenting practices, to enhance the development of EFs (Matte-Gagne and Bernier, 2011; Sarsour et al., 2011; Fay-Stammbach et al., 2014; Lengua et al., 2015).

Some research has shown that there are advantages of bilingualism on EF tasks involving conflict (Peal and Lambert, 1962; Bialystok, 1987, 2001, 2011; Bialystok and Martin, 2004; Bialystok et al., 2010; Poulin-Dubois et al., 2011; Barac and Bialystok, 2012; Garraffa et al., 2015; Antoniou et al., 2016; Blom et al., 2017; White, 2014, Unpublished). Conflict, in this context, is defined as a disagreement between two or more things, and it comes up whenever there are incompatible and competing responses or representations (Festman and Münte, 2012). Such tasks that present a conflicting situation include the dimensional change card sort task (DCCS). This task puts two pairs of rules in conflict with each other, and calls for children to pay attention to only one of them at a time (Bialystok, 1999).

Not all studies have shown a bilingual advantage on EF tasks (see review in Valian, 2015). Some recent studies found no differences in EFs between their monolingual and bilingual samples (Morton and Harper, 2007; Tare and Linck, 2011; Paap and Greenberg, 2013; Duñabeitia et al., 2014). In one study, the lack of difference held even when bilingual and monolingual children were carefully matched on age, gender, reading and mathematic skills, verbal and non-verbal IQ, family income, and the number of years of formal education of the parents (Antón et al., 2014). Other studies have found bilingual advantages only in particular age groups, such as preschoolers (Bialystok and Martin, 2004; Bialystok et al., 2006), children in middle childhood (Garraffa et al., 2015), young adults (Pelham and Abrams, 2014) or elderly adults (Craik and Bialystok, 2006; Bialystok et al., 2014). Other studies have not found a bilingual advantage in those age groups (see review in Valian, 2015).

One possible explanation for these variable findings is that there are individual differences among bilinguals that determine when an EF advantage is found (Festman et al., 2010; Valian, 2015). Indeed, some previous studies suggest that more balanced proficiency is associated with greater EF among bilinguals (Soveri et al., 2011; Yow and Li, 2015). The goal of this study was to investigate associations between bilingual children’s language dominance and use on the one hand and their EF abilities on the other.

### Language Dominance

One possible individual difference that could relate to bilingual children’s degree of EF is their language dominance. Most bilingual children display more advanced knowledge or proficiency in one of their two languages, otherwise known as their dominant language (Paradis and Nicoladis, 2007; Gathercole, 2016). Language dominance is a complex and multi-faceted construct (Silva-Corvalán and Treffers-Daller, 2016). In the present study, we focus on dominance as it relates to proficiency in the two languages and use a variety of measures to assess the children’s dominance. It is possible that more balanced bilinguals must reduce the interference from their other language more frequently than bilinguals with a strong dominance in one language. Indeed, some studies have shown that a more balanced knowledge in both languages leads to greater benefits on executive processes for bilinguals (Bialystok et al., 2006; Carlson and Meltzoff, 2008; Iluz-Cohen and Armon-Lotem, 2013; Tao et al., 2015; Antoniou et al., 2016; Blom et al., 2017; Thomas-Sunesson et al., 2018). For example, in a study comparing 6-year-old English-Italian bilinguals, English speakers with a limited knowledge of Italian, and English monolinguals, Ricciardelli (1992) found that the bilingual children performed significantly better than the other two groups in five of cognitive measures. Similarly, Bialystok and Majumder (1998) found that balanced bilingual children performed best on non-linguistic tasks that required control of attentional processing, even after controlling for age and language proficiency, when compared to the partial bilingual and monolingual groups. These patterns of results in the literature seem to suggest that the outcomes on cognitive performance may be dependent on the extent to which an individual is highly proficient in both languages.

Part of the reason that balanced bilingualism might be particularly strongly associated with cognitive advantages is the semantic organization of the mental lexicon. With increasing proficiency in both languages, bilingual children learn more translation equivalents (TEs) (Legacy et al., 2016). TEs refer to two words in different languages that refer to the same concept (e.g., *cake* and *gâteau*). Since both language systems are active in a bilingual’s mind (Grosjean, 1989), there could be competition between TEs when a bilingual uses one of the words. A higher proportion of TEs would therefore mean that bilinguals have to switch across the two systems and inhibit the irrelevant one more frequently, thereby enhancing their EF (Patterson and Pearson, 2004). In support of this argument, a recent study by Crivello et al. (2016) found evidence of enhanced EF mechanisms as a function of TE acquisition, where a bigger increase in the number of TEs predicted higher EF in toddlers through increased opportunities for switching across the two lexical systems.

Some studies have not found a relationship between balanced proficiency and enhanced EFs (e.g., Paap et al., 2014; Tao et al., 2015; von Bastian et al., 2016). For example, in a large-scale study comparing English monolinguals with simultaneous and early sequential Welsh-English bilinguals on a variety of EF tasks, no bilingual advantage was found (Gathercole et al., 2014). The authors attributed the lack of a bilingual advantage to the fact that their participants were all simultaneous or early sequential bilinguals. While this was a large-scale study and included participants from the age of 3 years through to adulthood, it is important to replicate. It is not clear why early onset of bilingualism would nullify a bilingual advantage, since early bilinguals, like late bilinguals, also show simultaneous activation of both languages even when processing only one (Nicoladis, 2006). Furthermore, some studies have shown EF advantages in both early and late bilinguals over monolinguals (Tao et al., 2011; Pelham and Abrams, 2014).

In sum, bilingual children’s language dominance may predict their EF. Specifically, the more balanced bilinguals might have more experience inhibiting interference from the other language than less balanced bilinguals. Inhibiting interference from the other language may be particularly important for TEs, where there is competing activation from a word in the non-target language. Not all studies have found a relationship between dominance and EFs. It is possible that the link between balanced proficiency and enhanced EF does not hold for early-onset bilinguals.

### Language Use

Language selection decisions are thought to be important for EF gains among bilinguals (Rubin and Meiran, 2005; Festman et al., 2010; Soveri et al., 2011). However, some studies of adult bilinguals have shown no relationship between language usage and EFs (Paap et al., 2014; von Bastian et al., 2016). In the studies with adults, a common measure is the frequency of usage of the two languages on a day-to-day basis.

In the present study, we measured how separately the bilingual children kept their two languages with monolingual interviewers in their two languages. While bilingual children can be shown to differentiate their two languages from early in development (Arnberg and Arnberg, 1992; Mishina-Mori, 2002; see review in Quay and Montanari, 2016), there is variability in how separately the languages of bilinguals are kept in use. While language separation might be at least somewhat related to language dominance, the two constructs can at least sometimes diverge. For example, a recent study showed that Chinese–English bilingual children could use as many different word types in their second language English as English monolinguals even though their proficiency in English was much weaker than that of the same-aged monolinguals (Nicoladis and Jiang, 2018). Furthermore, some bilinguals live in bilingual communities in which people use both languages with multiple people on an everyday basis, while other bilinguals live in more monolingual regions, requiring a greater degree of separation in use (Poplack, 1985; Ayeomoni, 2006; Baker, 2011; Gatt et al., 2016). In this study, we test whether children use a high degree of the target language in interviews in both of their languages.

One measure of the degree of separation in use is code-switching, or the use of two languages in a single unit of discourse (MacSwan, 2016). For example, a Spanish–English bilingual might say, *me voy al mall* ‘I’m going to the mall’, with most words in Spanish and the English word *mall*. In some studies, the frequency of code-switching among bilingual children has been found to be related to their proficiency (Nicoladis and Genesee, 1996; Ribot and Hoff, 2014; Yow et al., 2017), but not all studies have found the same directionality of that relationship. Ribot and Hoff (2014) showed that when the target language was Spanish, Spanish–English bilinguals in the United States used more English as their English proficiency increased. Thus, the children kept the languages less separate as their English proficiency increased. In contrast, for French–English bilingual children in Montreal, their use of code-switching largely reflected their proficiency in the target language, with higher proficiency in the target language being associated with lower use of code-mixing (Nicoladis and Genesee, 1997). In other words, the French–English bilingual children could have been code-switching to fill gaps in their knowledge in the target language. These results suggest that, in some communities, like Montreal, separation in language use may simply reflect a high degree of proficiency in both languages. In others, a high degree of proficiency in both languages could result in a low use of code-switching, particularly to the majority language of the community.

The community language practices could also be related to EFs in bilinguals. Tao et al. (2015) found a link between dominance and EF in Spanish–English bilinguals but not Mandarin–English bilinguals. Their study took place in Southern California where Spanish is a commonly used language. One explanation they considered for the different results among the two bilingual groups was that the groups differ on how frequently they have to monitor switches between two languages. Since there are many Spanish speakers in Southern California and many monolingual English speakers, Spanish–English bilinguals might have to do a lot of monitoring for which language is appropriate in a particular instance. In contrast, since there are few Mandarin speakers in Southern California, Mandarin–English bilinguals might have fewer instances of selecting an appropriate language. To our knowledge, the possible effect of the language community has not been taken into account in studies of EFs in bilingual children.

The present study was conducted with Canadian French–English bilinguals in both Edmonton, Alberta, and in Montreal, Quebec. In Montreal, many of its residents are fluent in both French and English (see Sioufi et al., 2016, for arguments classifying Montreal as part of Canada’s “bilingual belt”). Edmonton is a majority English-speaking city with a small francophone minority population (Aunger, 1999). Children’s language use shows effects of the linguistic community in the preschool years. For example, Genesee et al. (1995) showed that in Montreal French–English bilingual children’s code-switching, the use of two languages in a single unit of discourse, was related primarily to their dominant language (see also Nicoladis and Genesee, 1997). Specifically, the greater their proficiency in the language, the less likely they were to use words from their weaker language. In contrast, Paradis and Nicoladis (2007) showed that the code-switching among French–English bilingual children in Edmonton was affected not only by their stronger language but also by which language was the majority one. That is, both English-dominant and French-dominant children code-mixed infrequently in English (see also Ribot and Hoff, 2014). Paradis and Nicoladis (2007) argued that the Edmontonian children were sensitive to the fact that most French speakers also speak English while not all English speakers reliably speak French.

Bilinguals in Edmonton may therefore have to develop greater EF to avoid code-switching and interference from the non-relevant language on a daily basis. This enhanced practice in language control and language separation may in turn translate to larger EF advantages for the Edmonton bilinguals relative to the Montreal bilinguals.

In sum, bilingual children vary in how separate they keep their two languages in use. The degree of separation in use may be related to the children’s proficiency in their two languages as well as the community in which children live. If children keep their two languages separate in use, they may have greater EF than if they do not. Children living in an English-majority-language community like Edmonton might have greater practice keeping the two languages separate than children living in a bilingual community like Montreal. The children from a monolingual community might therefore show higher EF than the children from a bilingual community.

### Research Questions

The purpose of this study was to test language dominance and language use predictors of bilingual children’s cognitive flexibility. We included three measures of language dominance: (1) parental report, (2) relative vocabulary scores, and (3) TEs. The rationale for including three measures of dominance is that previous research has shown that different measures of dominance can yield different results (Bedore et al., 2012). We predicted that the more balanced the bilinguals and the higher rate of TEs, the higher their EF would be. We included two measures of language use: (1) linguistic community and (2) degree of language separation. We predicted that higher EF would be observed among the children in Edmonton (English majority-language community) relative to the children in Montreal (bilingual community) and among children who kept their languages separate in use relative to those who did not. These predictions are based on the assumption that the greater degree of experience inhibiting an inappropriate language, the greater executive control they would have to exercise on a daily basis, and therefore the more successful we expected them to be in the EF task.

The design of this study was correlational. Therefore, if we find the predicted correlations, we cannot identify directionality. We have phrased our research questions as if it is the experience with learning and separating languages that leads to enhanced EFs. However, it is equally possible that it is children with enhanced EFs who are more likely to become balanced bilinguals and separate their languages in use (Festman et al., 2010). We return to this point in the discussion.

## Materials and Methods

### Participants

The sample included a total of 62 French–English bilingual children, 36 from Montreal and 26 from Edmonton. The group comprised of 29 boys and 33 girls and had an age range of 46–82 months (*M* = 59.44, *SD* = 8.04). With regards to the age of exposure to both languages: 48 children were reported to have been exposed to both languages since birth, 10 between 1–2 years of age, and four between 2–4 years of age. The four bilinguals with age of onset to one language between 2–4 years were not outliers within the groups on any of our measures and so were included in all analyses.

The parents were asked: “Please choose the best description of your child’s French/English knowledge.” They were given five choices: (a) My child speaks French far better than English, (b) My child speaks French a bit better than English, (c) My child speaks both languages about equally well, (d) My child speaks English a bit better than French, and (e) My child speaks English far better than French. According to parental report, 18 were relatively balanced (i.e., chose option c; 11 were girls), 18 were slightly dominant (9 in French [option b] and 9 in English [option d]; 7 girls) and 25 were strongly dominant (11 in French [option a] and 14 in English [option e]; 14 girls). One parent did not respond to this question; this child’s data were excluded from the analyses including this measure.

### Procedure

The current study obtained approval from the institutional research ethics board. Parents signed an informed consent form, giving us permission to test their children. All children were asked for verbal assent before any tasks were carried out. The children completed a battery of language and cognitive tasks on different days for the two languages. We present the results only of the measures that are related to our research questions here. The order of the tests with a testing session varied according to the child’s engagement and comfort with the experimenter. As a default, the more passive tasks (such as the receptive vocabulary test) were administered earlier in the sessions than the more active tasks (such as the story-telling task). The order of the French and English sessions was counter-balanced. Different experimenters ran the sessions and both were native speakers of the testing language.

### Measures

#### Vocabulary

The Peabody Picture Vocabulary Test III (PPVT; Dunn and Dunn, 1997) and the Echelle de Vocabulaire en Images Peabody (EVIP; Dunn et al., 1993 – the French version of the PPVT) were used to measure children’s English and French receptive vocabulary size. Children had to respond to single words spoken aloud by the experimenter by either pointing to or indicating the number of the appropriate picture out of four black-and-white pictures. In accordance with PPVT standard starting and stopping criteria, the task started at children’s PPVT age set and stopped when children identified 8 or more items in a given set incorrectly.

The raw scores for the PPVT and EVIP are not on the same scale. For example, one 62-month old boy received a raw score of 91 on the PPVT and a raw score of 72 on the EVIP while his standard scores were virtually identical in the two languages (i.e., 118 on the PPVT and 120 on the EVIP). Therefore, in order to determine the child’s vocabulary dominance, we used the standard scores. For each child, we first noted in which language the standard score was higher. We then calculated the ratio of the standard scores (with the higher standard score for the child divided by the lower standard score for the child). Thus, the closer to one, the more balanced vocabulary scores a child showed. A higher ratio means that the children were showing greater unbalance in their vocabulary scores.

#### Language Separators

Children were shown a Pink Panther video, and then asked to recount as many details about it as they could after watching it. The children did this in both French and English. The total number of words in the stories told were calculated, including the number of French words used during the English session and the number of English words used during the French session (see **Table 1** for summary statistics). All the children but one used 92% or more words in one language or another in both sessions (the one exception used 81% French words in the French session). Given the categorical nature of children’s behavior, we classified the children as either language separators or not based on how much of the target languages the children used for both languages. To be classified as a language separator, a child had to use 92% or more French words in the French session and 92% or more English words in the English session. A child was classified as not being a language separator if his/her language use was less than 92% of the target language in one language. No child used less than 92% of the target language in both languages. Six children (4 balanced, 1 slightly unbalanced, and 1 unbalanced according to the parental report) were not included in this classification because they were not videotaped in one language or the other due to scheduling conflicts.

**Table 1:** Mean (SD) scores on the predictor variables by parental reports of dominance group.

	Balanced (*N* = 18)	Slightly dominant (*N* = 18)	Very dominant (*N* = 25)
Age in months	60.9 (7.0)	65.6 (9.3)	56.4 (4.8)
PPVT, standard scores	101.0 (14.5)	99.8 (15.1)	90.1 (30.6)
EVIP, standard scores	107.3 (17.1)	102.4 (17.4)	94.6 (24.4)
Vocabulary ratio	9.1 (7.3)	17.6 (17.0)	38.5 (32.3)
%Translation equivalents	23.5% (10.9%)	15.6% (12.6%)	8.7% (8.1%)
#Montreal/Edmonton	10/8	13/5	10/15
Language separator (# Yes/No)^†^	14/1	14/3	7/17
Average % Code-mixed words in English story	0% (0%)	0% (0%)	10.4% (31.1%)
Average % Code-mixed words in French story	0.2% (0.6%)	7.8% (23.1%)	44.8% (50.0%)

*PPVT, English receptive vocabulary; EVIP, French receptive vocabulary. ^†^Numbers do not necessarily total because of missing data (see text)*.

#### Translation Equivalents

The verbal semantic fluency task was used as a measure for children’s TEs. In this test, children were asked to name words from the following categories: clothes, animals, and food plus drinks. The given time per category was 30 s. The children did this in French during the French session and in English during the English session. The score obtained was a percentage of words that were TEs out of the total number of concepts generated. For example, if the child said “*cat, dog”* in English and “*chat, grenouille”* ‘cat, frog’ in French, the child generated a total of three concepts (cat, dog, and frog) with only one TE (for the concept cat). The ratio of TEs would be 1/3 = 0.333. We then multiplied by 100 to make a percentage.

Verbal semantic fluency tasks can measure both lexical knowledge and lexical retrieval (Weckerly et al., 2001) as well as executive control ability (Ruff et al., 1997; Shao et al., 2014). The total number of correct words generated is related to language ability, especially word knowledge, like vocabulary size (Ruff et al., 1997; Sergeant et al., 2002). The order in which words are generated can reflect executive functioning (Troyer et al., 1997; Hurks et al., 2010). In the present study we focused exclusively on the words generated rather than the order in which they were generated. This measure should therefore show strong correlations with the other dominance measures.

#### Executive Function Task

**FIGURE 1:**
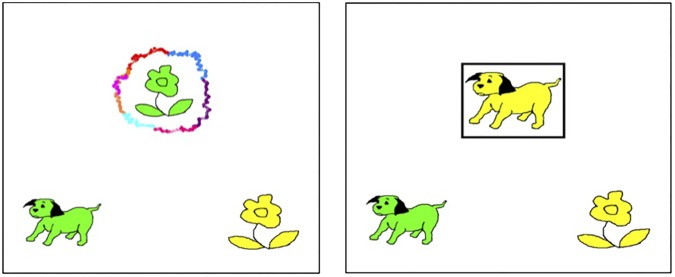
Examples of stimuli (**top**: yellow dog or green flower), response pictures (**bottom**: green dog and yellow flower), and visual cues (surrounding the stimuli) in each condition of the A-DCCS. Cues were the outlines of a multi-colored cloud and a black square. Children were instructed to pick the response picture that matched the stimulus on either color or shape, depending on the cue.

The Advanced Dimensional Change Card Sorting (A-DCCS) task (adapted from Chevalier and Blaye, 2009) was used as an index of participants’ EF. The task was run with E-Prime 2.0 software (Psychology Software Tools, Inc., 2007) and administered on a laptop computer during the English session. Children were asked to respond as quickly and accurately as possible by pressing either the “q” or “p” keys on the laptop keyboard, with the remaining keys covered and masked. In this computerized task, participants were required to match, based on task cues, a stimulus with one of two response pictures on either shape (Shape Game) or color (Color Game) on each trial. Stimuli were two pictures of different shapes and colors (a green flower and a yellow dog) and were presented at the top of the screen in the center (**Figure 1**). Each response image, a yellow flower and a green dog, matched the two stimuli on either shape or color. The two response images remained on the screen throughout the task and were presented on the two bottom corners of the screen and corresponding with the “p” and “q” keys respectively (**Figure 1**). Task cues surrounding the stimuli to indicate which game children should play were a multi-colored cloud (Color Game) and a black square (Shape Game; see **Figure 1**).

The A-DCCS consisted of three phases: color, shape, and mixed. Each phase started with a practice block followed by one test block for the color and shape phases, and two test blocks for the mixed phase. The experimenter provided children with help during practice blocks if necessary, but not during the test blocks. On every trial, the task cue and stimulus were simultaneously presented. Once a response was entered, the stimulus (without the task cue) would move onto the side of the given response. This was to simulate the traditional card version of the A-DCCS where cards are put into boxes, making the button-press response real for children. In the Color Game, children were instructed to press the key under the bottom (response) picture of the same color as the top (stimulus) picture. For the Shape Game, children were instructed to press the key under the response picture of the same shape as the stimulus. In the mixed phase, children were told that they would be playing both games at the same time. The mixed phase contained non-switch trials – where the relevant game recurs – and switch trials – where the relevant game changes.

The dependent variables for this task were the mixing and switching costs in accuracy, as well as reaction time for switching costs. However, it is important to note that previous research found a bilingual advantage in only the switching cost (Garbin et al., 2010; Prior and MacWhinney, 2010). We nonetheless include the mixing costs to verify the generalizability of the previous null findings. For all the reaction time measures, we removed the data for any child who responded two or more SDs slower than the average for that particular measure (between 3 and 5% of the data). Mixing costs compare performance on simple phases (just one relevant task/game across the entire block) with non-switch trials (the relevant task/game is identical to that of the previous trial) from the mixed phases (both tasks/games are relevant within the block; Chevalier and Blaye, 2009). Switching costs, on the other hand, compare switch trials (the relevant task/game is different from the previous trial) and non-switch trials within the mixed phases only. These are used in the literature as comparable to the bilingual process of juggling two languages: mixing costs reflect the task-decision process of goal setting and the difficulty of keeping two task sets activated; while switching costs reflect the switching process (Rubin and Meiran, 2005; Chevalier and Blaye, 2009). Goal setting is primarily reflected in mixing costs as it is present in the non-switch trials but not in the simple blocks (Chevalier and Blaye, 2009). As for the switching costs, both the non-switch and switch trials need goal setting, but only the latter require implementing a switch (Chevalier and Blaye, 2009). The dependent variables with accuracy were calculated using the following equations:

(1) Mixing costs in accuracy=Single accuracy−Mixed non−switch accuracy

(2) Switching costs in accuracy=Mixed non−switch accuracy−Mixed switch accuracy

We also included the reaction time for the switching costs, since Blom et al. (2017) found that some children showed a negative Flanker effect in reaction times.

## Results

Descriptive statistics for the predictor variables and the EF measures are summarized in **Table 2**, grouped by the parental report on the children’s dominance. The children in each dominance group were not equivalent on age (see **Table 1**), *F*(2,59) = 9.39, *p* < 0.001, $\eta_{p}^{2}$ = 0.241. LSD *post hoc* comparisons showed that all of the dominance groups differed from each other at *p* ≤ 0.049. The slightly dominant group was the oldest on average, followed by the balanced group, followed by the very dominant group. Given the age differences between groups, we partialled out age in presenting the main analyses in **Table 3**. As can be seen in **Table 2**, some children showed similar negative cost effect as reported in Blom et al. (2017), with many children responding faster to mixed switch trials than to mixed non-switch trials (*N* = 24).

**Table 2:** Scores on the advanced dimensional change card sort task by parental reports of dominance group.

	Balanced (*N* = 18)	Slightly dominant (*N* = 18)	Very dominant (*N* = 25)
Single accuracy	0.92 (0.12)	0.87 (0.12)	0.89 (0.09)
Mixed non-switch accuracy	0.81 (0.16)	0.77 (0.17)	0.77 (0.17)
Mixed switch accuracy	0.79 (0.13)	0.71 (0.17)	0.72 (0.15)
Single RT	1545.4 (596.4)	1470.6 (713.1)	1961.2 (998.1)
Mixed non-switch RT	2638.0 (1207.4)	2583.6 (707.9)	3782.0 (1255.4)
Mixed switch RT	2804.2 (1340.0)	3181.5 (1635.0)	4488.8 (1909.1)
Mixing costs in accuracy	0.09 (0.19)	0.10 (0.21)	0.19 (0.19)
Switching costs in accuracy	0.08 (0.12)	0.03 (0.12)	0.05 (0.14)
Switching costs in reaction time	26.9 (638.4)	382.5 (1900)	-4.1 (4824.3)

**Table 3:** Correlations between age, dominance measures, language use measures, mixing costs in accuracy, switching costs in accuracy, and switching costs in reaction times.

Variables	Parental dominance	Vocabulary ratio	TEs	Separators	City	Mixing costs	Switching costs	Reaction times
Age	-0.27*	-0.07	0.24	0.30*	0.02	-0.14	-0.06	-0.07
Parental dominance	-	0.50**	-0.52**	-0.57**	0.15	0.24	-0.07	-0.01
Vocabulary ratio	0.44^∗∗^	-	-0.32*	-0.27	0.18	0.00	-0.03	0.11
%TEs	-0.46^∗∗^	-0.34^∗^	-	0.50**	-0.14	-0.01	-0.14	0.06
Language separators	-0.55^∗∗^	-0.25	0.43^∗∗^	-	-0.17	-0.22	-0.05	-0.12
City	0.03	0.24	-0.09	-0.18	-	0.11	0.13	0.15
Mixing costs in accuracy	0.17	-0.02	0.01	-0.25	0.07	-	-0.25*	-0.25
Switching costs in accuracy	-0.06	-0.03	-0.08	0.02	0.15	-0.07	-	0.07
Switching costs in reaction times	-0.03	0.10	0.07	-0.14	0.15	-0.26^∗^	0.07	-

*Shaded cells (below the diagonal) show correlations with age partialled out. Parental dominance = parental report of dominance; %TEs = percent translation equivalents; language separators dummy coded so that 0 = no and 1 = yes; city dummy coded so that 0 = Montreal and 1 = Edmonton. Partial correlations for age below the diagonal. ^∗^P < 0.05, ^∗∗^P < 0.01.*

We had predicted that the parental reports of dominance, the vocabulary ratio, and the percentage of TEs (%TEs) would be converging measures of language dominance. The first-order correlations between the variables under study are summarized above the diagonal in **Table 3**. To include City as a correlate, Edmonton was coded as 0 and Montreal as 1. As can be seen in this Table, all the dominance measures are all highly intercorrelated in the predicted direction. For example, the negative correlation between parental dominance and TEs means that the more balanced the parents judged their child to be, the more TEs that child produced. One measure of language use (language separators) was also highly correlated with the dominance measures, such that the more balanced the children, the more likely they were to separate their languages in use. The other measure of hypothesized language use (City) was not related to any of the other variables. The language dominance and use variables did not correlate significantly with the mixing costs in accuracy, the switching costs in accuracy, or reaction times on the A-DCCS.

Below the diagonal in the shaded cells of **Table 3**, we present the correlations between variables, with age partialled out. It is the last three rows of this Table that present the data to address our research questions. None of the language dominance or language use measures was significantly correlated with mixing cost accuracy, switching cost accuracy, or reaction times for switching costs. In contrast, many of the language dominance and use measures remained significantly correlated with each other, even after controlling for age.

## Discussion

The purpose of the present study was to test whether language dominance and language use measures predicted the EF of French–English bilingual children. We used multiple measures of language dominance: parental report, the ratio of standardized vocabulary scores, and the percentage of TEs generated on a semantic verbal fluency task. We measured language use both by linguistic community (the English-majority-language community of Edmonton vs. the bilingual community of Montreal) and by whether the children separated their languages by the language of the interlocutor. We predicted that the children who were relatively balanced in their bilingual abilities would perform better on the EF task than the unbalanced bilinguals (Ricciardelli, 1992; Bialystok and Majumder, 1998). The task-decision process that happens in the mixed-task blocks resembles the bilingual situation where decisions of which language to use have to be made in conversations, and smaller mixing costs reflect better control of attentional processing and higher ability in keeping two different task-sets active (Braver et al., 2003; Soveri et al., 2011; Brocki and Tillman, 2014). Consequently, there is reason to expect that balanced bilinguals have more experience in having both languages activated and paying attention to non-salient features of input (Bialystok and Majumder, 1998).

Our results showed no relationship between either language dominance or language use measures and children’s EF as indexed by mixing or switching costs in accuracy or reaction times for switching costs. These results contrast with those of some previous studies, showing larger EF advantages for balanced bilinguals (e.g., Ricciardelli, 1992; Bialystok and Majumder, 1998; Crivello et al., 2016). In some previous studies, a bilingual advantage has been shown in only switching costs (Garbin et al., 2010; Prior and MacWhinney, 2010). In this study, we found no correlation between either switching or mixing costs and language dominance or use.

One possible reason for the lack of relationship between the language dominance and use measures with EF is that the participants in the present study were either simultaneous or early sequential bilinguals. Recall that Gathercole et al. (2014) argued that they did not show a bilingual advantage because their participants were simultaneous or early sequential bilinguals. We think this is an unlikely explanation for two reasons. First, other studies with early-onset bilinguals have shown advantages (Bialystok and Majumder, 1998). Second, early bilinguals also show simultaneous activation of both of their languages (Grosjean, 2010).

Another possible reason for the null findings is that we did not have enough statistical power to show a significant relationship between the language measures and EFs. A power analysis showed that we have 80% power to detect a correlation of 0.35 (two-sided), suggesting that we do have adequate power. Also, other studies showing positive effects have sometimes included smaller or equivalent sample sizes than the ones here (e.g., Crivello et al., 2016; Thomas-Sunesson et al., 2018). Furthermore, studies including even larger sample sizes have shown null effects (e.g., Gathercole et al., 2014).

A third, and we expect the most likely, possible explanation for our results is the following. If there are individual differences between bilingual children that predict the degree of EF, language dominance and use may not be valid predictors. Other researchers have raised other possible variables that could contribute including socioeconomic status (Morton and Harper, 2007; cf. Kang et al., 2016), immigration status (de Bruin et al., 2015), culture (Kang et al., 2016; cf. Barac and Bialystok, 2012), working memory capacity (Namazi and Thordardottir, 2010), and others (see Donnelly et al., 2015).

If this explanation is correct, then there is growing evidence that the rationale behind predicting a bilingual advantage in EF needs to be reconsidered. Note that the present study was not designed to test whether there is a bilingual advantage, an issue that has been addressed extensively elsewhere (e.g., Paap and Greenberg, 2013; Paap et al., 2015; Sorge et al., 2017). Rather, the purpose of this study was to test whether the degree of language dominance or separation in use predicted EF. The rationale for a bilingual advantage has been that the experience selecting the appropriate language for the context and inhibiting the inappropriate language would lead to general EF advantages. We found no evidence for this claim (consistent with some other studies; Gathercole et al., 2014; Paap et al., 2014; von Bastian et al., 2016).

We noted at the outset of our study that our correlational design does not allow us to distinguish the directionality of effects (or lack of effects). Some researchers have argued that bilinguals with high EF ability may be the ones who become highly proficient in both languages and/or learn to separate the languages well (e.g., Festman and Münte, 2012). While we see no evidence for this interpretation in our present study, we should also point out that our study was not designed to test that prediction. Some studies have found a bilingual advantage in older children but not younger children (e.g., Garraffa et al., 2017; although cf. Gathercole et al., 2014). A better design to test the possibility that high EFs lead to balanced proficiency and use would be a longitudinal one.

Before closing, we would like to draw readers’ attention to one unexpected finding. The linguistic community (either monolingual Edmonton or bilingual Montreal) was not correlated with any of the other measures of language dominance and use. In contrast, the other measures of language dominance and use tended to be highly intercorrelated. Recall that we had predicted that Edmonton bilinguals would be more likely to keep their two languages separate in use than Montreal bilinguals. One possible reason for this finding is that an individual child’s linguistic community may vary from the larger community (see Byers-Heinlein et al., 2017, for discussion specifically about children’s individual language communities in Montreal). Previous studies have shown that family language practices can affect bilingual children’s language dominance and use (e.g., Altman et al., 2014; see review in Quay and Montanari, 2016). Future research can test for that possibility.

## Conclusion

The present study showed no relationship between bilingual children’s cognitive flexibility and language dominance/use. These results, in combination with others, raise doubts as to the rationale usually given for a purported bilingual advantage in EF. To the extent that there is a bilingual advantage in non-linguistic EF tasks, it may not be because of experience selecting and inhibiting languages alone, at least within the age range we examined here. Other researchers have raised other possibilities, including language proximity (e.g., Antoniou et al., 2016; Garraffa et al., 2017; cf. Antón et al., 2014) or task specificity (see review in Valian, 2015) or developmental changes (Garraffa et al., 2017).

## Ethics Statement

This study was carried out in accordance with the recommendations of Tri-Council policy of Canada. The protocol was approved by the Research Ethics Board of the University of Alberta. The parents or legal guardians of all participants gave written informed consent.

## Author Contributions

EN supervised data collection, helped with data analysis, and helped write the manuscript. DH ran the first analyses, wrote the first draft of the manuscript, and provided feedback on the penultimate draft. SW provided the appropriate EF measure and contributed to the analyses and write-up of the manuscript.

## Conflict of Interest Statement

The authors declare that the research was conducted in the absence of any commercial or financial relationships that could be construed as a potential conflict of interest.
